# Early Mortality Syndrome Outbreaks: A Microbial Management Issue in Shrimp Farming?

**DOI:** 10.1371/journal.ppat.1003919

**Published:** 2014-04-24

**Authors:** Peter De Schryver, Tom Defoirdt, Patrick Sorgeloos

**Affiliations:** Laboratory of Aquaculture & *Artemia* Reference Center, Department of Animal Production, Ghent University, Gent, Belgium; The Fox Chase Cancer Center, United States of America

A recent disease of farmed Penaeid shrimp, commonly referred to as “early mortality syndrome” (EMS) or more technically known as “acute hepatopancreatic necrosis disease” (AHPND), was first reported in southern China in 2010 and subsequently in Vietnam, Thailand, and Malaysia [1]. The EMS/AHPND disease typically affects shrimp postlarvae within 20–30 days after stocking and frequently causes up to 100% mortality. The Global Aquaculture Alliance [2] has estimated that losses to the Asian shrimp culture sector amount to USD 1 billion. The causative agent of EMS/AHPND has been reported to be a bacterium—more specifically a pathogenic *Vibrio* belonging to the Harveyi clade, presumably *Vibrio parahaemolyticus* [3]. So far, this has been the only description of a bacterial isolate capable of causing EMS/AHPND. Strategies to remedy this disease are urgently needed. However, as long as it remains unclear whether or not all incidences of EMS/AHPND are caused by one or more specific *V. parahaemolyticus* strains, approaches that focus on controlling the presence or activity of vibrios in general have the highest chance of decreasing the risk of EMS/AHPND outbreaks.

We argue that the proposed strategy of total disinfection of pond bottom and water to kill possible vectors of EMS/AHPND [1] may contribute to the epidemic spread of the EMS/AHPND disease rather than control it, and that microbial management strategies may be the key to minimizing the risk of EMS/AHPND outbreaks. We suggest stocking shrimp postlarvae in systems with a mature microbiota (such as algae-rich greenwaters and microbially matured water systems), as environments primarily colonized by slow-growing harmless bacteria might best guarantee the prevention of EMS/AHPND outbreaks.

The ecosystem disturbance caused by the current practice of disinfecting ponds to remove potential pathogens or their carriers prior to stocking shrimp postlarvae most probably does more harm than good. The increase in nutrient availability after disinfection combined with a destabilized and impoverished microbial community (and a consequent lack of competition) favors fast-growing bacteria (such as many pathogenic *Vibrio* spp.) in recolonizing the environment [4]. Considering that EMS/AHPND most probably is caused by a *Vibrio*, this practice is thus more likely to stimulate proliferation of the EMS/AHPND-causing agent in the pond than counteract it. In fact, important lessons can be learned from the outbreaks of luminescent vibriosis in the early 1990s. This disease was caused by *Vibrio harveyi* and closely related species—all belonging to the Harveyi clade of vibrios (and therefore closely related to the causative agent of EMS/AHPND) [5]. Luminescent vibriosis occurred during the first 10–45 days after stocking of shrimp postlarvae in the grow-out ponds. The outbreak of the disease was found to be preceded by a substantial increase in the number of opportunistic vibrios in the pond water [6], and this increase followed pond disinfection and was associated with a perturbed microbial community in combination with the presence of nutrients [6], [7].

In the last year, several remedies to control EMS/AHPND—mostly based on empirical observations—have been proposed on public discussion lists. It was for example stated that EMS/AHPND is less prevalent in ponds colonized by copepods (small crustaceans used as live feed for the larvae of aquaculture animals). Copepod presence is an indicator of a naturally mature/stable ecosystem, as it requires constant amounts of phytoplankton and bacteria as feed [8]. Alternatively, using greenwater technology has also been related with a lowered incidence of EMS/AHPND in practice. Greenwater systems (in contrast to clear water systems) are characterized by a mature micro-algal and bacterial community and have been shown before to result in decreased *Vibrio* levels and decreased animal mortality [9], [10]. Several mechanisms have been linked to the beneficial effect of greenwaters, including the algal production of antibacterial substances [11] and compounds that inhibit virulence gene regulation (e.g., quorum sensing inhibitors [12]). However, we think that the bacteria that are associated with the micro-algae should not be neglected either, as they might be able to compete with pathogens for available nutrients and to produce compounds affecting viability and/or activity of pathogens [13].

Similar to greenwater technology, microbially matured water systems have been developed to minimize the presence of pathogens that are able to grow fast and are consequently capable of quickly invading “empty” niches. The microbial maturity of water can be described based on the ecological theory of *r*/*K* selection [14]. Microbially matured water is characterized by a dominance of slow-growing bacteria with a limited nutrient supply per bacterium, the so-called *K* strategists. They eliminate the niches for fast growing bacteria, the *r* strategists, which include many disease-causing *Vibrio* spp. [4]. As such, *K* selective pressure in shrimp postlarvae culture systems may avoid proliferation of the vibrios causing EMS/AHPND. *K* selective pressure in grow-out ponds can be achieved by minimizing disturbances leading to sudden variations in nutrient levels in the rearing water during shrimp culture in order to keep nutrient supply per bacterium constant and low, and by colonizing the influent water with nonpathogenic bacteria and/or algae at a carrying capacity close to that of the rearing water [15]. In case pond disinfection is applied as a hygienic barrier, maturation of the pond water after flooding should be ensured prior to stocking, in order to prevent *r* strategists from dominating the system.

It needs to be stressed that the mature ecosystem approach aims at preventing EMS/AHPND and will not act as a curative treatment for EMS/AHPND-infected shrimp. In order to cure affected animals, one should develop and apply techniques that result in minimal disturbances of the nontarget microbiota in order to be compatible with microbially mature systems (Box 1). Finally, one should make sure that the larvae used for stocking are EMS/AHPND-free (e.g., by applying microbial management practices in hatcheries as well).

**Box 1. EMS/AHPND Mitigation Strategies Should Be Compatible with Microbially Mature Ecosystems**

The use of antibiotics and/or disinfectants would not only destroy microbially mature systems, it has also proven ineffective in treating diseases caused by luminescent vibrios (i.e., *V. harveyi* and closely related bacteria, which are closely related to the bacteria causing EMS/AHPND), and alternative methods have been proposed in this context [5]. Antivirulence therapy, i.e., disarming the pathogens (by affecting the expression of virulence genes) rather than killing them, could be a promising approach in this respect. Interestingly, we recently reported that the use of compounds that inhibit quorum sensing (bacterial cell-to-cell communication regulating the expression of virulence factors) significantly decreased the mortality of giant river prawn larvae caused by pathogenic *V. harveyi* [16]. Alternatively, biological agents that are only active at the site of infection (i.e., the gut) could be applied. Poly-β-hydroxybutyrate is an example of a natural compound that upon depolymerisation interferes with the energy metabolism of pathogenic cells, and it has been shown to control *Vibrio* presence in the gut of giant river prawn larvae [17]. However, unfortunately, these kinds of therapies are still in the research phase.

In conclusion, the recent outbreaks of EMS/AHPND suggest that modern intensive shrimp farming practices need to be critically reviewed. We argue that microbial management practices (including growing animals in microbially mature ecosystems and applying biocontrol strategies that are compatible with these systems) are currently largely neglected, even though they are a key factor in solving these problems.
